# Embedding professional development within the curriculum of graduate programs: An impact survey from biomedical departments in a faculty of medicine

**DOI:** 10.1371/journal.pone.0321207

**Published:** 2025-04-02

**Authors:** Helen Miliotis, Nana Hyung-Ran Lee, Rachael Cayley, Christina Zakala, Allan Kaplan, Reinhart Reithmeier

**Affiliations:** 1 Department of Physiology, Temerty Faculty of Medicine, University of Toronto, Toronto, Ontario, Canada; 2 Department of Biochemistry, Temerty Faculty of Medicine, University of Toronto, Toronto, Ontario, Canada; 3 Department of Immunology, Temerty Faculty of Medicine, University of Toronto, Toronto, Ontario, Canada; 4 Centre for Graduate Professional Development, School of Graduate Studies, University of Toronto, Toronto, Ontario, Canada; 5 Department of Pharmacology and Toxicology, Temerty Faculty of Medicine, University of Toronto, Toronto, Ontario, Canada; 6 Department of Psychiatry, Temerty Faculty of Medicine, University of Toronto, Toronto, Ontario,; 7 Institute of Medical Science, Temerty Faculty of Medicine, University of Toronto, Toronto, Ontario, Canada; University of Manouba Higher School of Commerce of Tunis: Universite de la Manouba Ecole Superieure de Commerce de Tunis, TUNISIA

## Abstract

Although the career pathways of biomedical PhDs are increasingly diverse, graduate education continues to rely upon an apprenticeship model, whereby supervisors train the next generation of researchers. To broaden the core competency skills for research productivity and career development across the Temerty Faculty of Medicine’s departments and institutes, a for-credit curricular Graduate Professional Development (GPD) course has been implemented as an integral part of the graduate curriculum in multiple departments. We used three key principles to advance this integrated professional development curriculum: i) a train-the-trainers method for implementation, ii) direct student engagement via a students-as-partners model, one-on-one consultations and videos, and iii) a faculty development program. While programming of this sort has direct benefits, less is known about how students themselves experience curricular GPD. To address the gap in understanding the impact, research was undertaken to inquire about students’ perceived skills development and attitudes towards career choices after taking the GPD course. According to our findings, students see the curricular GPD course as a valuable complement to their graduate education and feel more prepared for their next steps in their career exploration. With buy-in from students, faculty and academic leaders, embedded curricular GPD has transformed the academic culture of graduate education in our faculty, helping our students with skills that empower them to be prepared for their own career development to meet the competitive career realities of our time.

## Introduction

Doctoral graduate education is historically modelled after an apprenticeship-mentor relationship for graduate preparation into academic careers; in recent years, however, we have increasingly seen a broader range of career outcomes and an overall rethinking of graduate education. These shifts are due in part to the increasing number of PhD holders; the number of PhD graduates in Science, Technology, Engineering, Mathematics (STEM) in the United States has almost doubled since the early 1980s (~19,000 in 1982 and ~ 36,000 in 2011) while the number of academic positions remain the same at approximately 3,000 per year [1].

Recent international trends suggest that many doctoral graduates do not work in traditional academic careers. Career outcome studies in the United States have shown that 39% of STEM doctoral graduates [2] and 25% of biomedical sciences doctoral graduates [3] are employed as faculty. The findings that most PhD graduates are not in academic positions are reflected in many regions, including the United Kingdom [4], Australia [5], and others worldwide [6]. These trends are the same in Canada: the University of British Columbia has seen 26% of its PhDs enter the tenure track [7], while the 10,000 PhDs Project at the University of Toronto showed that 18% of PhD graduates in life sciences who graduated from 2000 to 2015 were tenure-track professors in 2016, with an additional 29% in the post-secondary sector, including post-doctoral training [8].

Familiarity with these trends has led to a broader awareness that PhD holders find employment outside of academic settings [1,2]. Considerations around rethinking graduate education and reform began decades ago [9] and continue to be discussed in the higher education literature today [10–15]. One of the most prominent themes in the doctoral education literature concerns graduate student competencies and employment [16]. PhD holders may experience increased competition for academic positions or a need for additional skills outside of those mastered during their degree to transfer to non-academic careers [11].

These findings support the argument that providing core competency skills training is essential for students if they are to explore, acquire and succeed in careers both outside or within the academy. This training can be informed by career development learning (CDL) frameworks that support student knowledge, self-management and skills around careers and the role that higher education institutions play in career development learning [17].

The offerings around career development vary across institutions. Although graduate professional development (GPD) programs do exist at many universities, these are typically a series of co-curricular (not for credit, extracurricular) workshops as opposed to curricular programming. Co-curricular GPD has several notable drawbacks. For example, students may decide not to participate due to a perceived lack of time and a supervisory culture that does not fully support co-curricular activities. In addition, having purely co-curricular programming can also lead to a lack of coherence between workshop offerings, an absence of peer community formation, and a gap in discipline-specific career considerations. In light of these challenges, there are calls to consider embedding curricular professional development into graduate programs [18,19].

Career development programs have been implemented at many institutions through the NIH-BEST program [20] and embedded curricular professional development can be implemented through various approaches. One approach is the integration of skills such as scientific communication into content specific existing courses [21,22]. Another strategy is to embed career exploration, individual development plans (IDPs), workshops, courses and other professional development activities at each year of the graduate degree [23,24]. This has allowed for further acceptance and campus-wide trust of GPD and allowed for further expansion of related co-curricular activities [23].

A few stand-alone courses in career exploration and professional development do exist [25–27]. In one case study of a career exploration and planning course, authors report gains on the course outcomes at the end of the course on one very small cohort of students (n = 12) as reported through course surveys [25]. In another career exploration course, student course evaluations showed gains in career exploration skills and increased considerations of alternative careers beyond academia, while another career planning course that was also open to post-doctoral fellows also showed gains in career management self-efficacy [26]. These studies suggest that graduate students benefit from career exploration and planning courses. While these programs and studies add valuable insights into the effect of these courses on graduate student training, more research is needed to better understand their efficacy and impact.

Based on an early awareness of the need for more GPD structured training, the Department of Biochemistry at the University of Toronto launched a for-credit graduate professional development GPD course for graduate students in 2012 [28] to further support professional skills development during their degrees. In this paper, we report on the implementation of the integrated curricular GPD course across the department and eventually, across the faculty that also led to further faculty-wide GPD programming.

Our work not only describes the implementation of a stand-alone course in professional development but also aims to assess the efficacy beyond course evaluations. We are using research ethics approved surveys on multiple cohorts of students to study not only the skills our students gain but also their preparedness and attitudes towards their next steps to begin to address some of the research gaps in this field, as there is still a “striking lack of data” ([23] p.877) that tests the efficacy of various graduate professional development approaches. In fact, while many opinion and descriptive pieces on GPD are increasingly being published, there is a gap in studies on the actual implementation and impact of research stream GPD [29]. There are calls for more research in doctoral reforms, including on how to better support success in doctoral education, and in ways that academic frameworks can support the careers of doctoral students within and beyond academia [11]. Our study will help to address some of these research gaps and contributes to the better understanding of programming for our graduate students.

In what follows, we describe the program history of faculty-wide GPD initiatives, including the GPD course development and other related offerings in departments across the Temerty Faculty of Medicine, detailing our approach. To ensure that this innovative programming was grounded in empirical evidence, we also present research findings on the impact of curricular career support on doctoral student training. Our data suggests that students feel more prepared for their next steps post-graduation after participating in curricular GPD and view the course as a valuable complement to their graduate training. Finally, we discuss the implications of our findings for enhancing graduate education programming.

## Program history of faculty wide initiatives

### The graduate professional development (GPD) course

The original Biochemistry GPD syllabus was created after consultations with graduate alumni, faculty, and graduate students; the result was a pilot course in Fall of 2012 with PhD students in the Department of Biochemistry. The course is aimed at empowering graduate students with skills in 1) self-reflection using the *Science Careers IDP* [30], 2) creating meaningful engagement with these reflections and 3) communication of marketing their science and themselves. The Individual Development Plan (IDP) framework encourages them to engage with career planning and effective scientific productivity during graduate school and to develop their core competency skills. The IDP reflection provides a foundation in which students can create meaningful engagements, or interactions with their community of research or non scholarly interest which strengthen their development. Communication skills were enhanced through the mediums of the three-minute thesis presentations (3MT) and various job application techniques like informational interviews, résumé writing and mock interviews. The course model is generative, with six, 2-hour sessions, with concepts from each class building on the previous one (Table 1). A more detailed class syllabus is provided as Supporting Information (S1 Generic GPD Syllabus). These three skills were highlighted to develop based on feedback from previous course evaluations and alumni who had entered the job market.

**Table 1.** Generative programming for GPD curriculum-embedded course illustrating the development of skills in oral and written communications networking.

In-Person Class Times	Writing Skills Development (Job Applications)	Career Planning, Research Productivity and Networking Skills Development	Oral Communications Skill Development
I	Students find and bring in their own job description to discuss requirements and how to cater towards the job	LinkedIn Profile and IDP Discussion	Preview video on storytelling and three-minute thesis (3MT) before class. Discuss and practice how to give a one-minute pitch “tell us about yourself.”
II	Discussion of CAR (context, action, results) statements on a résumé	How to find a career professional to request informational interview	Slide karaoke (impromptu presentation of a never-before seen slide) for peer feedback and then try another one right after with feedback implementation
III	Peer feedback on students’ résumés or CVs	Student report back any problems finding guests – discuss other strategies	Bring in a script for the 3MT for peer feedback
IV	Peer feedback on students’ cover letters, submit job application to instructor	Discussion on virtual and conference networking	Prepare a 3MT slide for peer feedback
V	Instructor discusses job application submissions	Discussion on mentorship and sponsorship	Practice 3MT presentations with peer feedback
VI	Student may resubmit after editing for additional feedback	Guest Networking Event	Give final 3MT presentations with judges

Since the winter of 2020, the model of delivery of the course has been a flipped classroom; students are sent readings and short videos [31] to preview and class time is used for discussion and peer feedback in breakout groups [32].

### Faculty wide GPD interventions

Following the successful implementation of this course in a single department of Biochemistry, it was implemented in other departments at the Temerty Faculty of Medicine. Since each department has a unique culture and curriculum, we used three key principles for GPD to be adopted across the whole faculty.

1. A one-on-one “train-the-trainer” method was applied to allow instructors to bring the course content to the Departments of Immunology [33], Molecular Genetics, Pharmacology-Toxicology, Physiology [34], and the Institute of Medical Sciences. Instructors either shadowed the Biochemistry GPD course and/or received individual training for syllabus content and assessment structure and have coordinated or are now coordinating their own GPD courses. Some GPD content such as an introduction to the IDP, email professionalism, networking, principles in conflict management and setting expectations were taken by faculty to be included in mandatory first year graduate courses in Biochemistry’s Scientific Skills for Biochemists [35] and Scientific Skills for Immunologists [33].
2. For those students who were in departments without their own GPD course, we aimed to increase student engagement by providing interdepartmental workshops and student one-on-one consultations (approximately 50 per year) on ways to effectively use the IDP for career development and research productivity. Guidance on mentorships programs, professional development workshops and/or one-on-one consultations have been implemented for these students as well. To promote peer-to-peer mentorship, we also hosted or continually host a sharing of best practices of peer graduate student mentorship from most departments at an annual workshop.
3. A monthly faculty workshop series was implemented from 2016-2022 [36] to inform faculty members more broadly about various GPD topics (IDP, mentee-driven mentorship, conflict management, emotional intelligence, strategic planning, equity diversity inclusion (EDI), design thinking) from expert speakers (S2 Faculty Development). This series included effective practices on supervision and mentorship with over 76 faculty participants (with 100% finding the workshops helpful based on feedback forms). This workshop has now been transformed into a yearly faculty-wide symposium with sharing of challenges and wise practices. Engaging faculty directly has enhanced buy-in from those members of the faculty who previously thought of GPD as simply a co-curricular activity.

From its inception, the GPD program at the Temerty Faculty of Medicine has been highlighted in a number of reports [37–42] for its innovative approach to graduate education. While the benefits of the programming were being recognized, there was a gap in understanding how students themselves feel about these professional development opportunities. In the sections that follow, we describe the research we undertook to better understand student perceptions of the skills gained through curricular GPD and their attitudes towards their future steps.

## Methods

### Ethics statement

To comply with ethical standards of research, the work was conducted under the University of Toronto’s Human Participant Ethics Protocol (RIS 38711) and approved by the University of Toronto Research Ethics Board (REB) on 10/01/2020. Written information regarding the study and contact information for the principal investigators and the research ethics protocol were listed when the survey link was emailed. Consent was via completion of the survey. Those that did not consent were not required to complete the survey.

### Data collection and student demographics

To assess the impact of the GPD course on students, anonymous surveys were conducted during the recruitment period of 10/01/2020 to 07/12/2023 to ten cohorts of the course. Study participants were graduate students enrolled in the GPD course (average class size around 15) offered through the departments of Biochemistry, Immunology, and Physiology. For the pre-survey, 121 responses were collected, of which 26 students were enrolled in an MSc degree, and 95 were enrolled in a PhD. For the post-survey, 97 responses were collected, of which 18 students were enrolled in an MSc degree, and 79 were enrolled in a PhD. Questions were designed to measure how students felt about taking the course, their perceptions on the skills/knowledge gained and if their attitudes changed about their next steps. Open-ended questions were included for students to further express their thoughts and to provide general feedback on the course. The survey was circulated to GPD-enrolled students before the course started (pre-survey) and after the course ended (post-survey). The responses were collected anonymously through Microsoft Forms and reported as aggregate.

### Data analysis

Anonymous student survey responses were collected and analyzed by an independent research student. The post-survey questions around skill gains were reported as a mean (1: no gain – 5: very large gain) and a corresponding standard error of the mean (SEM). The survey questions around perceptions on next steps were reported as a mean (1: strongly disagree – 5: strongly agree), and a corresponding standard error of the mean (SEM). Differences between the anonymous two survey responses for perceptions on next steps were analyzed with an independent two-tailed t-test assuming equal variance at a significance level of α =  0.05 to compare the mean differences between survey answers.

## Results

### The GPD course is positively received by students

For the first iterations of the course between 2016–2019, informal anonymous class feedback forms were used for course improvements. Interestingly, 100% of student respondents found some or all of the contents of curricular GPD helpful to their graduate education, with 100% recommending it to their peers (233 respondents). While we could see student enthusiasm about the course, we lacked an in-depth understanding of why it was received so well and its impact. These questions prompted us to move beyond course feedback forms to a formal REB approved research study to better understand and evaluate student perceptions of the skills gained through curricular GPD and their attitudes towards their future steps.

Between 2020–2023, students from Biochemistry, Immunology and Physiology that took the curricular embedded GPD course stated that 98% would recommend this course to other students (n = 97), and 98% agreed that this course is a good complement to their graduate studies (n = 97). These findings are in line with our previous feedback from students. Subsequent sections of the survey reveal deeper insights into the student experience.

Our survey asked students to report on their perceived gains in their knowledge and skills based on course topics, and the results are summarized in Table 2. The largest gains were reported in résumé/cover letter skill development for any job application regardless of sector. Students were particularly enthusiastic about realizing the transferable skills gained during a biomedical graduate degree to a variety of future opportunities. Students also reported gains in their professional online presence and their ability to utilize these tools for networking. Furthermore, while students are often aware of careers within academia, they reported large gains in knowledge of careers outside academia enabled by class discussions, guest speakers, and informational interviews. Overall, all the topics that students were asked about resulted in student gains.

**Table 2:** Student perceived gains on their knowledge and skills after taking the GPD course reported as a mean (1:no gain – 5: very large gain), Post-survey (n = 97).

	Post Survey Gains	
Topic	MEAN	SEM
Resume and cover letter skills	4.24	0.0913
Knowledge on careers outside academia	4.15	0.0959
Professional online presence	3.86	0.1190
Oral communication for a general audience	3.66	0.0960
Networking	3.63	0.0976
Strategies to get a job faster upon graduation	3.61	0.1168
Written Communication for a general audience	3.56	0.1067
Understanding the importance of work life integration and wellbeing	3.56	0.1125
Knowledge on careers in academia	3.35	0.1218
Organization skills to reduce time to completion	2.53	0.1198

These results demonstrate that the GPD course was perceived by most students as adding value to their graduate programming - this can be seen in the following student comment:

*“My graduate degree is about more than developing my research skills, and it is worth spending the time and energy during my degree to fulfil other experiences that will prepare me for future success.”*

The notion that investing the time into GPD was worthwhile was echoed in many other student comments, such as:

*“I also realized that I really need to work on, and set aside time for, career planning and professional development activities while I am still a student.”*

Finding the time for these activities can be challenging, but the structured course format with a defined timetable and topics was welcomed by students:

*“I have been wanting to focus on professional development, but never set aside the time to do so. Taking this course helped me work towards those goals, with like-minded people, during designated time periods and helped me really start thinking about what the next two years of my degree will look like.”*

Furthermore, the foundations of GPD may inspire students to undertake further co-curricular programming that is offered at the institution, increasing uptake in those activities. Many students expressed further interest in related topics, such as in the following comment:

*“I will keep an open mind for opportunities that come my way. But I will take these skills from the class as much as I can and try to actively search for more opportunities.”*

Another benefit of course-based GPD is the resulting cohort structure. Many of our graduate students are in laboratories across different buildings and may not have many chances to meet and connect with other graduate students across our departments. Taking the GPD course as a cohort becomes an experience that students take together, resulting in community building and fostering connections. The following comments echo some of these sentiments.

*“… I also really appreciated taking this course with my cohort. It was a nice opportunity to re-connect and remind myself that most people in my cohort are experiencing the same worries as I am, therefore we should reach out to each other more and discuss the issues we are all experiencing as we think about our future careers.”*

and

*“This course was very reassuring in the fact that my anxieties over career development post-grad are shared amongst many of my peers and allowed me to connect with my peers on significant topics and life events outside of academia.”*

Our survey continued to explore the student experience by asking statements around their feelings and attitudes about their next steps after graduation, both before and after the course. Our results show changes in student attitudes after taking the course and are shown in Table 3. As seen in Table 3, students report feeling much more prepared for their next steps upon graduation. Preparedness was mentioned in many student comments, such as the following:

**Table 3:** Changes in attitude around next steps after taking the GPD course reported as a mean (1:strongly disagree – 5:strongly agree). Pre-survey (n = 118–121), post-survey (n = 97).

	Pre		Post		
	Mean	SEM	Mean	SEM	*p-value*
1. I feel prepared for my next steps upon graduation.	2.61	0.087	3.70	0.088	<0.0001
2. I believe this course will help me be successful in my career, no matter what it is.	3.88	0.075	4.21	0.075	0.003
3. The skills I gain in graduate school will be useful both in academic and non-academic careers.	4.28	0.068	4.49	0.061	0.022
4. Thinking of my future life fills me with hope.	3.43	0.090	3.72	0.091	0.023
5. I am excited about future possibilities.	3.90	0.084	4.13	0.081	0.048

^a^*p*-values calculated by two-tailed T-test.

*“Being more equipped to plan for my career after taking the course is very empowering. I feel much more prepared to work on professional development than I was before GPD.”*

Furthermore, more students believe that the course, as well as the skills gained during graduate school will be beneficial to both academic and non-academic careers – this suggests a shift in awareness of their transferable skills.

Interestingly, we also show insights into the overall effects of the course on student mindsets. Our results show that more students report feeling more hopeful about their future and agree that they are more excited about future possibilities. There were many comments that implied more positive outlooks after completing the course; here are some representative ones:

*“Although I am still uncertain of what the future holds beyond graduate school, I am more excited than I was previously about the possibilities that await. I feel confident that there are many paths I may take and many different versions of a successful career.”*

and

*“I don’t know what my occupation will be after the PhD. This does not worry me, I’m on the contrary excited about all the possibilities that I will have and hopeful that I can do something I love.”*

And finally

*“It (the course) made me feel more at ease regarding my next steps and made me feel quite hopeful.”*

Open ended questions around future possibilities show that while academia was the top perceived career option before the course, after the course students reported much more variability in potential career options. These included careers in industry, communications, and more – shifting to the actual careers that PhD outcomes data has reported [8].

## Discussion

Our research shows that our students reported that they benefited from curricular GPD. The course added value to their graduate education by improving career readiness skills and positively contributing to their attitudes around their next steps after graduation. Students saw the value of enhancing their skill set and building their professional network as key steps in exploring diverse career options and feeling more prepared for their next steps after graduation.

Moving GPD from co-curricular to curricular for-credit courses has resulted in a number of benefits, including a culture shift in graduate education, a stand-alone course code with a stepwise course progression, adjusting for skills needed for career preparation, expanding the student population who engages in professional development, and an embedded faculty member to help mentor students throughout their years in graduate school.

Embedding curricular GPD across a multi-departmental faculty has caused a cultural shift regarding graduate education in both students and professors, highlighting the critical importance of transferable skills and networking in the development of diverse career pathways for PhD students. Taking a course with a progression of topics and associated learning objectives can allow students the time and space to explore different discipline specific career options, develop their core competencies and learn to effectively communicate them. A designated course code emphasizes the pedagogical value of the course and can alleviate some of the pressures students may feel from their supervisors when engaging in professional development. Having a core instructor throughout the course results in each student receiving more effective and personalized guidance for their next steps for the year they are taking the course and throughout their studies.

Depending on the graduate program, there is often a mismatch between what activities students report as valuable for job preparation and what they actually do [43]. Endorsing a curricular component exposes students early on to different options and guides them in goal setting, potentially steering them into further workshops at the graduate school, career center, or research institute that are more aligned to their goals. In this way, departments/faculties can introduce discipline specific career outcomes but then endorse other institutional offerings, thus leveraging existing resources. In our experience, the curricular endorsement may also increase uptake in other related offerings that usually only a subset of students self-select to engage in.

Integrating GPD into the curriculum also increases the chance that more students be introduced to these important ideas. The students that would benefit the most from professional development offerings may be the ones that are not very engaged to begin with. Providing those students with professional development courses can expose them to the benefits and encourage them to take a more active part in their professional goal setting and competency development. These skills are not only going to help them in career planning, but during graduate school as well. For example, they can communicate their science more effectively to different audiences, maximize collaboration opportunities at conferences through improved networking skills, and essentially become more effective “ambassadors” of their research group.

We suspect other potential advantages to departmental curricular programming. Although we have no data yet to support these, our observations suggest that curricular GPD can lead to:

I) forming student communities of peer mentorship of professional and research development within their discipline, whether as entering cohorts or during their graduate program.
II) incorporating individual development plans (IDPs) within graduate programs and courses to emphasize their importance to both faculty and students [44].
III) applying the skills discussed in classes to other peer student initiatives such as wellness, Equity, Diversity and Inclusion (EDI) initiatives, conflict management and mentorship.
IV) departmental research-faculty buy-in and support in the notion of diverse career development for graduate students.

Any decision to integrate GPD into the curriculum must consider the implications for doctoral completion times. Interestingly, some research has shown that engaging in professional development activities during a biomedical PhD does not seem to increase time to degree completion [45] or affect manuscript output [46]. Indeed, course work promotes multi-tasking and time management in research-intensive biomedical graduate programs. Enabling the opportunity to engage in professional development and exposing students to various career possibilities can motivate them as they plan their next steps into either academic or non-academic careers.

Our findings also suggest that students may feel more hopeful and excited about their next steps after taking the course. Does GPD programming have an impact beyond simply career development, perhaps positively contributing to one’s overall outlook? It would be interesting to consider future research into resilience, self-efficacy, and hope.

While we have now seen how enthusiastic graduate students are about curricular GPD, we need to know more about the alumni perspective, after they move into the workforce. Do they think GPD still helps them beyond the classroom into promotions and career development? Our next steps will be to assess the value and impact of these departmental GPD programs, through a post-graduation survey of alumni. The literature tells us that when alumni from STEM PhD programs were asked what professional development would have been helpful based on their own experiences, the top suggestions were i) career path awareness and preparation, ii) networking opportunities, and iii) designated course/time for professional development [43]. Our GPD offerings cover those areas, and we will survey our alumni for their feedback. As part of such an initiative, diverse alumni will also be interviewed to provide compelling narratives of their career paths to inform current students of the various careers open to them. Although course feedback and case studies with testimonials by alumni do exist, now that we have more graduates from the GPD program, a more thorough assessment of graduates would inform GPD program leaders regarding curricular topics to add or improve to continue to better prepare our students for their future steps.

We demonstrate that our embedded curricular graduate professional development (GPD) course and faculty wide initiatives towards graduate career skills and awareness have had a positive impact on our student skillsets and mindsets towards career development. Rethinking our graduate education initiatives can better prepare our students to navigate future career transitions. While our work has focused on biomedical doctoral students, the framework presented here of embedded curricular professional development can be used as a model for other units to adopt across different disciplines. Our programming can be successfully adopted by other departments and faculties when thinking about how to modify their graduate student offerings and faculty wide promotion of career awareness.

The GPD course started as a pilot in one department and has been successfully replicated across several departments. This has generated a culture shift that has permeated throughout the Temerty Faculty of Medicine by students and faculty embracing GPD as an essential, integrated component of the graduate curriculum. This emphasis on curricular GPD is empowering the next generation of biomedical researchers to reflect on their growth as scientists and explore diverse career options that will allow them to become our next scientific thought and action leaders.

## Supporting information

S1 File Generic GPD syllabus. (PDF)

S2 File Faculty Development.
(PDF)

S3 File Data.
(XLSX)

## References

[1] Schillebeeckx M, Maricque B, Lewis C. The missing piece to changing the university culture. Nat Biotechnol. 2013;31(10):938–41. doi: 10.1038/nbt.2706 24104758

[2] Turk-Bicakci L, Berger A, Haxton C. The nonacademic careers of STEM PhD holders. American Institute for Research; 2014.

[3] Mathur A, Cano A, Kohl M, Muthunayake NS, Vaidyanathan P, Wood ME, et al. Visualization of gender, race, citizenship and academic performance in association with career outcomes of 15-year biomedical doctoral alumni at a public research university. PLoS One. 2018;13(5):e0197473. doi: 10.1371/journal.pone.0197473 29771987 PMC5957427

[4] Hancock S. The employment of PhD graduates in the UK: What do we know?. 2020. Available from: https://www.hepi.ac.uk/2020/02/17/the-employment-of-phd-graduates-in-the-uk-what-do-we-know/

[5] McCarthy P, Wienk M. Advancing Australia’s knowledge economy: Who are the top PhD employers?. The University of Melbourne; 2019.

[6] OECD. Doctorate holders. 2015.

[7] The University of British Columbia. UBC Phd career outcome survey. 2017. Available from: https://outcomes.grad.ubc.ca/.

[8] Reithmeier R, O’Leary L, Zhu X, Dales C, Abdulkarim A, Aquil A, et al. The 10,000 PhDs project at the University of Toronto: Using employment outcome data to inform graduate education. PLoS One. 2019;14(1):e0209898. doi: 10.1371/journal.pone.0209898 30650157 PMC6334897

[9] Zumeta W. Doctoral programs and the labor market, or how should we respond to the “PhD glut”?. Higher Education. 1982;11(3):321–43. doi: 10.1007/BF00155622

[10] Osborne BJ, Carpenter S, Brunett M, Rolheiser C, Korpan C. Preparing graduate students for a changing world of work: Editor’s introduction. Can J High Educ. 2014;44(3):i–ix. doi: 10.47678/cjhe.v44i3.186033

[11] Sarrico CS. The expansion of doctoral education and the changing nature and purpose of the doctorate. High Educ. 2022;84(6):1299–315. doi: 10.1007/s10734-022-00946-1

[12] Enders J. Border crossings: Research training, knowledge dissemination and the transformation of academic work. High Educ. 2005;49(1–2):119–33. doi: 10.1007/s10734-004-2917-3

[13] O’Shea S, Jungblut J. Foreword. Stud High Educ. 2023;48:1517–8. doi: 10.1080/03075079.2023.2253030

[14] Teelken C, Andreasen KE, Galimberti A, Rasmussen A. An international exploration of post-PhD careers. Discussing the issues of employability and intersectorial mobility. Stud High Educ. 2023;48(10):1519–22. doi: 10.1080/03075079.2023.2253031

[15] Leshner AI, Scherer L. Critical steps toward modernizing graduate STEM education. Issues Sci Technol. 2019;35:46–9.

[16] Cardoso S, Santos S, Diogo S, Soares D, Carvalho T. The transformation of doctoral education: a systematic literature review. High Educ. 2022;84(1):885–908. doi: 10.1007/s10734-021-00805-5

[17] Watts A. Career development learning and employability. 2006.

[18] Fuhrmann CN, Fenn SL, Hidalgo D, Horowitz BB, Loring HS, Nayak S, et al. Chapter 13 - Creating the “new normal”: Career development embedded into the Ph.D. curriculum for all trainees. In: Infante Lara L, Daniel L, Chalkley R, editors. BEST.: Academic Press; 2020. pp. 171–181. doi: 10.1016/B978-0-12-820759-8.00013-9

[19] Sinche M, Layton RL, Brandt PD, O’Connell AB, Hall JD, Freeman AM, et al. An evidence-based evaluation of transferrable skills and job satisfaction for science PhDs. PLoS One. 2017;12(9):e0185023. doi: 10.1371/journal.pone.0185023 28931079 PMC5607200

[20] Lenzi RN, Korn SJ, Wallace M, Desmond NL, Labosky PA. The NIH “BEST” programs: Institutional programs, the program evaluation, and early data. FASEB J. 2020;34(3):3570–82. doi: 10.1096/fj.201902064 31960495

[21] Heustis RJ, Venkatesh MJ, Gutlerner JL, Loparo JJ. Embedding academic and professional skills training with experimental-design chalk talks. Nat Biotechnol. 2019;37(12):1523–7. doi: 10.1038/s41587-019-0338-1 31796928

[22] Brownell SE, Price JV, Steinman L. Science communication to the general public: why we need to teach undergraduate and graduate students this skill as part of their formal scientific training. J Undergrad Neurosci Educ. 2013;12(1):E6–10. 24319399 PMC3852879

[23] Fuhrmann CN. Enhancing graduate and postdoctoral education to create a sustainable biomedical workforce. Hum Gene Ther. 2016;27(11):871–9. doi: 10.1089/hum.2016.154 27762630 PMC5116696

[24] Furhmann C. Career development in graduate education. Iss Sci Technol. Available from: https://issues.org/forum-42/.

[25] Branan J, Li X, Wheeler R. Building a career planning course for STEM PhDs. Nat Biotechnol. 2018;36(12):1217–9. doi: 10.1038/nbt.4312 30520859

[26] Layton RL, Solberg V, Jahangir AE, Hall JD, Ponder CA, Micoli KJ, et al. Career planning courses increase career readiness of graduate and postdoctoral trainees. F1000Res. 2020;9:1230. doi: 10.12688/f1000research.26025.2 33163161 PMC7605208

[27] Norman-Burgdolf HL, Vanderford NL. Preparing future professionals by enhancing workforce readiness. Nat Biotechnol. 2016;34(1):111–3. doi: 10.1038/nbt.3459 26744984 PMC5500181

[28] Lee N, Reithmeier R. A graduate course in professional development. Science. Oct, 1. Available from: https://www.science.org/content/article/graduate-course-professional-development.

[29] Van Wart A, Djorić D, D’Silva NM, Layton R, Hardy L, Suelzer E, et al. An emerging field: An evaluation of biomedical graduate student and postdoctoral education and training research across seven decades. PLoS One. 2023;18(7):e0282262. doi: 10.1371/journal.pone.0282262 37490486 PMC10368290

[30] Furhmann C, Hobin J, Lindstaedt B, Clifford P. myIDP Science Careers. 2023. https://myidp.sciencecareers.org/

[31] Anonymous. Graduate professional development videos. 2021. Available from: https://www.youtube.com/playlist?list=PL095D5AmPAaop0BwdEAcCnsKPp-KH5-Bd

[32] Biochemistry UoT. Graduate professional development BCH2201. Biochemistry UoT. 2023. https://biochemistry.utoronto.ca/courses/bch2201/

[33] Immunology UoT. IMM2200H Graduate professional development. 2023. Available from: https://immunology.utoronto.ca/imm2200h.

[34] Physiology UoT. Graduate professional development course. 2023. Available from: https://physiology.utoronto.ca/graduate-professional-development-course.

[35] Biochemistry UoT. BCH2101H – Scientific skills for biochemists. 2023. Available from: https://biochemistry.utoronto.ca/courses/bch-2101h/.

[36] Anonymous. GLSE Faculty Development Series. 2022. Available from: https://rhse.temertymedicine.utoronto.ca/best-practices-graduate-supervisors. .

[37] Denecke D, Feaster K, Stone K. Professional development: Shaping effective programs for STEM graduate students; 2017.

[38] McIntyre C. Are there too many PhDs? Turns out, maybe not: A look at where PhDs end up after leaving the Ivory Tower. National Post. June 2. Available from: https://nationalpost.com/news/canada/are-there-too-many-phds-turns-out-maybe-not-a-look-at-where-phds-end-up-after-leaving-the-ivory-tower

[39] Fung H. What comes next?. The Varsity. 2016. [cited 2023 November 21]. https://thevarsity.ca/2016/02/21/what-comes-next/

[40] Vanhouten A. Professional development program teaches valuable soft skills to science PhDs. University Affairs. Available from: www.universityaffairs.ca/career-advice/career-advice-article/professional-development-program-teaches-valuable-soft-skills-science-phds.

[41] Bell E, Miliotis H, MacEachern L, Longo L, Karatzas C, Brady A. Insights and perspectives from the PhD to employee forum. CJCD. 2021;20:94–104. doi: 10.53379/cjcd.2021.95

[42] Council of Canadian Academies. Degrees of success: The expert panel on the labour market transition of PhD graduates. 2021.

[43] Ganapati S, Ritchie TS. Professional development and career-preparedness experiences of STEM Ph.D. students: gaps and avenues for improvement. PLoS One. 2021;16(12):e0260328. doi: 10.1371/journal.pone.0260328 34914698 PMC8675721

[44] Gould J. Career development: A plan for action. Nature (London). 2017;548(7668):489–90. doi: 10.1038/nj7668-489a

[45] Mathur A, Chow CS, Feig AL, Kenaga H, Moldenhauer JA, Muthunayake NS, et al. Exposure to multiple career pathways by biomedical doctoral students at a public research university. PLoS One. 2018;13(6):e0199720. doi: 10.1371/journal.pone.0199720 29933412 PMC6014666

[46] Brandt PD, Sturzenegger Varvayanis S, Baas T, Bolgioni AF, Alder J, Petrie KA, et al. A cross-institutional analysis of the effects of broadening trainee professional development on research productivity. PLoS Biol. 2021;19(7):e3000956. doi: 10.1371/journal.pbio.3000956 34264929 PMC8282014

