# Supplementary material for: Embedding professional development within the curriculum of graduate programs: An impact survey from biomedical departments in a faculty of medicine
Source: PLoS One. 2025 Apr 2;20(4):e0321207. doi: 10.1371/journal.pone.0321207 (PMC11964202; doi:10.1371/journal.pone.0321207)
Supplement: S1 File — (PDF) [file pone.0321207.s001.pdf]

BCH2201H Graduate Professional Development (GPD)

Course instructor | Nana Lee | nana.lee@utoronto.ca   Credit: 0.25

Welcome to GPD!

You are about to embark on an adventure. GPD empowers learners to optimize their graduate experience to prepare for any career path. The primary goals of this course are to 1) reflect on your skills, interests, and values, 2) explore options, 3) learn how to set a professional development plan during your studies and effectively market yourself during and after graduate school. Being part of the GPD team also grants you lifetime office hours.

Reading

Success in Graduate School and Beyond by Nana Lee and Reinhart Reithmeier, 2024. You may pick it from the U of Toronto bookstore in the Medical Books, reference section, or order it online through U of T Press, Amazon or Indigo. *IDP* refers to the individual development plans from Science Careers. https://myidp.sciencecareers.org/

Course outline

Class	Prework	Topics to discuss	To bring	Deliverables
**Class 1**	Start reading the book, update IDP, view videos 1-3, 5 (10 min each)	Individual Development Plans<br>Options	Updated SMART goals	
**Class 2**	Videos 6-7	Brainstorming Your Meaningful Engagement<br><br>informational Interviews<br><br>Reading a Job Description<br><br>Script Feedback	Your Job description<br><br>Your 3 minute script	
**Class 3**	Videos 8-9<br><br>Videos 10-12	Q & A on Networking<br><br>Group activity on CARs	Job Application with CARs	Job description, cover letter, resume
**Class 4**	Video 13<br><br>Video 14	Mock Interviews<br><br>Presenting Impromptu<br><br>Peer Feedback on slide	One static slide	Job description, cover letter, resume

**Class 5**	Videos 15, 17-20	Q & A on videos<br><br>3MT practice	Revised static slide	
**Class 6**	Video 21	3MT final presentation, networking	Final slide	3MT presentation, IDP & book reflection, informational interview presentation (if applicable)

Breakdown of Marks (deliverables + presentations + attendance/participation)

1. Written Assignments (50%)

Job application: Write a one-page cover letter and a resume for a job description or proposal. Include the job description as well. **(25%)**

IDP and book reflection: Write a 700-900 word reflection (not a summary or review) on "Success In Graduate School and Beyond" and how it changed your views, if any, on designing your own graduate experience and/or career path. Also include a reflection on your IDP with three specific SMART goals. **(25%)**

2. Presentations (25%)

Three Minute Thesis (3MT) final: Present your research in three minutes with one static slide to the general public, describing why it is significant to today's society. (12.5%)

Guests: Present your informational interview summary or bring in a guest for last class. (12.5%)

3. Attendance and Participation (25%)

Rubrics and Suggestions

Deliverable #1. Job application - Cover Letter/Resume (10 points each, 20 points in total)

Criteria – Cover Letter	Points
Company header and date	1
Opening paragraph	2
Middle section (WHY that company, what are you offering)	4
Last paragraph/closing	1
Signature	1

Your contact info	1

**Criteria – Resume**	**Points**
Overall appearance	2
Experience – CAR statements	6
Education	1
Skills if applicable, selected awards/publications	1

Deliverable #2. Book Reflection (20 points in total)

**Criteria**	**Points**
Insightful new ideas regarding your own career development and reasonable SMART goals with specific next steps	10
Clarity, grammar, professionalism	10

Presentation #1. Three Minute Thesis (20 points in total)

**Criteria**	**Points**
Clarity	5
Time (between 2.5 minutes to 3 minutes)	5
Helpful illustration	5
Engagement	5

Pointers

1. Start with a story or a question. Here is a good example. "Do you know that your own immune cells fight cancer? Some of you may be walking around with cancer cells (pause) but are perfectly healthy because your own immune cells can overcome them."

2. Do not use filler words such as "like, so, uhm, ok, right, uh." If you feel compelled to use them, just leave a slightly longer pause.

3. End with a pause and then "Thank-you."

4. Slide has a title on top, your name on the top right hand corner. No PIs, no institutions, no degrees.

5. Every word and picture should be pointed out and talked about. You should refer to your slide within the first 20 seconds, if not earlier, and continue on with the illustrations as visual aids. Minimize text.

6. Be confident, own the room with your story and your presence. You are the expert.

7. Best way to practice is to draw the slide as you tell the story like a chalk talk.

8. Abandon the notes and use cue words on the slide. A good series of illustrations will bring forth the story.

Presentation #2. Networking Event

All students are to contact a professional who has the career you would perhaps explore after graduate school. Guests should be somebody who has been through the job application, interview process, landed their job and are now working professionals who are being remunerated for their work. Sources of potential guests: LinkedIn with keywords of your alumni department, network of your labmates, 10,000 Coffees, LSO, LSCDS.

You (not a friend) are to invite them to this networking event. If they do not respond in 2 weeks, ask with a gently reminder and if they do not write back, do not contact them again. Try to find someone else. Please email me your confirmed guest's name, degrees, job title and affiliation for the Networking Event.

If your guest cannot make it for this date, request an informational interview. Please prepare a two-minute presentation to summarize your informational interview experience during Class #6. Topics to include are how you contacted them, how they made their career transition, and what you thought of the whole experience. Enjoy the process!

Individual Development Plan Consults

As part of GPD, we have a 30 minute one-on-one consult to discuss anything you like regarding your own GPD and to go over an IDP.

I will be sending out a doodle to book everyone's meetings – please only check ONE timeslot when you can make it and make sure nobody else has signed up for that time.

Resources

Video links listed on the course website.

https://biochemistry.utoronto.ca/courses/bch2201/

Resume CARs

http://careerrocketeer.com/2012/06/what-does-a-car-have-to-do-with-your-resume.html

Resume Action Verbs

https://gecd.mit.edu/sites/default/files/jobs/files/resume-action-verbs.pdf

Core Competencies

http://www.workforce.com/2002/09/03/31-core-competencies-explained/

Syllabus copyright 2020 by Nana Lee. Instructors are welcome to use in whole or in part of the syllabus with permission by Nana Lee.
