# Supplementary material for: Embedding professional development within the curriculum of graduate programs: An impact survey from biomedical departments in a faculty of medicine
Source: PLoS One. 2025 Apr 2;20(4):e0321207. doi: 10.1371/journal.pone.0321207 (PMC11964202; doi:10.1371/journal.pone.0321207)
Supplement: S2 File — Faculty Development. (PDF) [file pone.0321207.s002.pdf]

Temerty Faculty of Medicine
Faculty Development Workshop Series coordinated by Dr. Nana Lee

A series of workshops designed to optimize your supervision of trainees so they are fully prepared to succeed during and after graduate school. Presented by expert speakers from academia and the biotech industry. Some workshop topics include: graduate professional development, individual development plans, strategic communications, self-marketing, leadership, conflict resolution, experiential learning.

Intended for 1) those who currently have or are planning to train graduate students; 2) those interested in establishing their own graduate professional development workshop series, course, or program for their department;

I. Introduction: Why GPD for Graduate Students?

This workshop gives an introduction to how graduate professional development programs can help trainees strengthen core competency skills for a successful PhD completion and with career goals.

Topics to cover are 1) the core competency skills which are to be developed during the PhD, 2) where current PhDs are employed, 3) the 30+ career options available for PhDs in the life sciences, 4) the skills needed to develop for the first job acquisition and further career development in the academic and non-academic sector, 5) skills required to create their own jobs.

II. Individual Development Plans (IDPs) Part I

Many universities in the USA have implemented IDPs as part of the graduate curriculum with financial supports via BEST grants from the NIH. IDPs are well established in industry, with some top companies holding five IDP meetings a year with each professional. In this workshop, faculty will become familiarized with the IDP, discuss some case studies of the IDP and how to use its contents each year with changing goals to better prepare students for their thesis project management and professional development.

III. Individual Development Plans Part II

Even with committee meetings and PACs in place, we are witnessing students with longer times to completion (SGS data). How do we prevent these? What are the resources at U of T that could be used by the PI and/or student to help facilitate a TTC of 5 years? This workshop is a continuation of part I, with faculty discussion on how to implement the IDP to guide the student to a 5-year PhD.

IV. Helping Students Develop Strategic Communications I
This workshop discusses how faculty can help student with the methods of communications during graduate school with student seminars, conferences, teaching, three minute thesis, communicating with non-experts and lay audience. These transferable communication skills are essential for success during graduate school and with the career thereafter.

V. Helping Students Develop Strategic Communications II: Self-Marketing
This workshop will the cover strategic networking and marketing transferable skills acquired during the PhD. Discussions will cover methods of informational interviews, professionalism in social media, LinkedIn, meetings, follow-ups. This workshop also discusses how faculty could help students with strategic communications so that they are able to effectively pitch their ideas to key opinion leaders in academia, the industry, business investors, and fellow innovators with little science background.

This second half of the workshop will cover case studies of students marketing themselves effectively for non-academic positions. After discussing example career paths and relevant skills, participants will learn and apply a resume technique suited for non-academic positions. Faculty will be coached on how to guide students in translating their experience into an attention-grabbing resume that is aligned to their target job.

VI. Supporting Student in Developing Leadership and Creativity
The PhD provides the foundation to foster scientific, critical thinking skills, problem-solving and analytical skills. Along with these core competencies, students should be developing leadership in scientific thought in their research field and in controlling their own career action plans. Some also develop team leadership. This workshop discusses ways to help students develop leadership and creativity to help them become the innovators and leaders of Canada’s knowledge economy.

VII. Helping Students with Conflict Resolution, Organizational Navigation and Emotional Intelligence
Many PhD trainees will have little or no experience with these concepts before they enter graduate studies. Most will have some introduction to these ideas during graduate school training, but are overwhelmed or bewildered. In this workshop, we will discuss methods of conflict resolution which faculty can implement or help support their students with laboratory personnel relations. An introduction of emotional intelligence and navigating an organization in which faculty can use themselves and teach their own students will be discussed. We will also discuss how the IDP can be used to optimize honest communications with the supervisor.

VIII. Helping Students with Experiential Learning Opportunities
Many of the first jobs landed by PhDs are those in which they have connections within the company or after a rewarding experiential learning opportunity, as an intern or volunteer. We

will discuss how faculty are able to support students in finding and creating these opportunities with case studies and guest speakers with direct experience.

IX. Mentorship Matters

This workshop gives an overview of best practices in mentorship to optimize student engagement from an academic and industry perspective. Highlights include: ideas to encourage mentee-driven mentorship, considering diversity and unconscious bias when mentoring, mentorship program models. Feel free to bring your ideas and programs to share.

X. Tools for Student Engagement

This workshop discusses some tools to use for student engagement with graduate research, communications, and self-development into the "whole scientist." Some tools to discuss are the Science Careers individual development plan, iBiology's Planning Your Scientific Journey, growth mindset theory, crafting a meaningful life, the power of why.

XI. Conflict Management and Student Wellness

Student productivity is affected by conflict and wellness. How do we try to prevent conflict at the workplace and how do we manage when distress arises?

XII. Entrepreneurship

How do you encourage entrepreneurial and intrapreneurial skills so your graduate trainees can create their own collaborations with other academic or industry partners? How do you build these partnerships into potential funding opportunities? How might students or postdoctoral fellows from your lab start companies on the side? How might you start one?

XIII. Highlights of Graduate Professional Development (GPD) in One Workshop

This workshop reviews the essential highlights of curriculum-embedded GPD and how the individual PI can use GPD to increase research productivity and professional development.

XIV. Best Practices in Reducing Times to Completion

What are some of the methods used by current graduate coordinators to address time to completion? What impact do these methods have? This discussion will be led by graduate coordinators from various departments at the Faculty of Medicine.
